# Associations of Perchlorate, Nitrate, and Thiocyanate with Bone Mineral Density in the US General Population: A Multi-Cycle Study of NHANES 2011–2018

**DOI:** 10.3390/nu16162658

**Published:** 2024-08-11

**Authors:** Donglan Wang, Ying Zhang, Yayu He, Fengmei Song, Yan Tang, Limou Chen, Yangcan Wang, Fei Yang, Xueqiong Yao

**Affiliations:** 1 School of Public Health, Hengyang Medical School, University of South China, Hengyang 421009, Chinayayu_he@163.com (Y.H.);; 2 Hunan Provincial Key Laboratory of Clinical Epidemiology, Department of Social Medicine and Health Management, Xiangya School of Public Health, Central South University, Changsha 410000, China; 3 Laboratory of Ecological Environment and Critical Human Diseases Prevention of Hunan Province, School of Basic Medical Sciences, Hengyang Medical School, University of South China, Hengyang 421009, China

**Keywords:** perchlorate, nitrate, thiocyanate, bone mineral density, national health and nutrition examination survey

## Abstract

Background: Perchlorate, nitrate, and thiocyanate are widely recognized as endocrine disrupting chemicals, which are closely related to thyroid function. Animal and human studies show a correlation between thyroid hormone and bone mineral density (BMD). However, it remains unknown whether perchlorate, nitrate, and thiocyanate were associated with BMD. This study aimed to explore the association between perchlorate, nitrate, and thiocyanate exposure with BMD. Method: A cross-sectional analysis among 5607 participants from the 2011–2018 National Health and Nutrition Examination Survey (NHANES) was conducted in the present study. Perchlorate, nitrate, and thiocyanate were detected in urine by ion chromatography. Survey-weighted generalized linear regression, restricted cubic splines, and qgcomp models were used to assess the association of BMDs with single and mixed perchlorate, nitrate, and thiocyanate exposures. In addition, age, gender, and BMI stratified these associations. Results: Negative associations were found between perchlorate and nitrate with BMDs. Furthermore, based on the qgcomp model results, the combined association of perchlorate, nitrate, and thiocyanate exposure was negatively associated with BMDs (β = −0.017, 95% CI: −0.041, −0.024 for total BMD; β = −0.017, 95% CI: −0.029, −0.005 for lumbar BMD). Additionally, there was a significant effect after gender, age, and BMI stratification between perchlorate, nitrate, and thiocyanate with BMDs in the normal weight group (β = −0.015, 95% CI: −0.020, −0.011 for total BMD; β = −0.022, 95% CI: −0.028, −0.016 for lumbar BMD) and children and adolescents group (β = −0.025, 95% CI: −0.031, −0.019 for total BMD; β −0.017, 95% CI: −0.029, −0.005 for lumbar BMD). Conclusions: The present study indicated a negative correlation between BMDs and urinary perchlorate, nitrate, and thiocyanate levels, with nitrate being the main contributor to the mixture effect. People with normal weight and children and adolescents were more likely to be affected.

## 1. Introduction

Osteoporosis (OP), a progressive skeletal disorder, is characterized by diminished bone mineral density (BMD) and microstructural degradation [1,2]. From 2017 to 2018, the age-adjusted prevalence of osteoporosis among individuals aged 50 years and older was recorded at 12.6% [3]. Osteoporosis-related fractures pose a significant clinical and economic burden. An estimated 2 million incident osteoporotic fractures at a total cost of USD 17 billion occur each year in the United States [4]. BMD is the most widely used parameter for assessing bone strength and commonly used to diagnose OP [5]. BMD increases rapidly during childhood and adolescence and peaks around 20 years old [6]. The preferred sites for measuring BMD from adolescence into old age are the total body and lumbar spine [7]. A meta-analysis study has demonstrated that people with decreased hip BMD may be more vulnerable to hip fractures (the most serious type of osteoporotic fracture) [8]. Therefore, identifying the factors influencing BMD is critical for preventing OP and improving quality of life. In addition to well-known factors affecting BMD, such as smoking, lack of exercise, poor diet quality, and certain medications, exposure to pollutants in the environment can also be relevant to BMD [9,10,11]. Therefore, it is essential to explore the environmental pollutants associated with BMD extensively.

Perchlorate, in addition to its natural occurrence, is widely utilized in military applications, explosives, and fireworks, and its detection rates in drinking water and food are very high [12,13]. Nitrate, as a precursor to nitrite, is a part of the natural nitrogen cycle and is commonly used as a fertilizer in the agricultural industry and a preservative in the food industry, particularly in processing meats [14]. Thiocyanate, a biomarker for cigarette smoke exposure, is also found in cruciferous vegetables (like radishes and kale) as well as dairy products [15,16]. Previous studies have shown that these substances are ubiquitous in food and the environment, leading to widespread human exposure [17]. Since these three chemicals are primarily excreted in urine, urine samples are commonly used as biomarkers to assess human exposure and potential health effects [18].

Perchlorate, nitrate, and thiocyanate are recognized as inhibitors of the sodium/iodide symporter (NIS) due to their ability to exert endocrine-disrupting effects by affecting thyroid function [19]. Recent epidemiological studies have shown an association between exposure to perchlorate, nitrate, and thiocyanate and disrupted thyroid hormone levels [20,21]. Furthermore, several studies have reported important links between thyroid hormone levels and hypertension, diabetes, and cancer [12,22,23]. However, there is growing evidence linking central sensitization of thyroid hormones to perchlorate, nitrate, and thiocyanate exposure [21], whereas impaired sensitivity to thyroid hormones may be a contributing factor to abnormal BMD [24,25]. Thus, it is necessary to investigate the relationship between BMD and exposure to perchlorate, nitrate, and thiocyanate.

However, to our knowledge, no epidemiological studies have yet examined the relationship between co-exposure to these three substances with BMD. A study in China suggested that nitrate was negatively associated with bone strength [26]. In addition, a placebo-controlled trial study indicated that organic nitrate was not correlated with postmenopausal women’s BMD [27]. Nevertheless, the relationships between BMD and perchlorate, nitrate, and thiocyanate in single and mixed exposures are largely unknown.

Here, we examined the relationships between urinary perchlorate, nitrate, and thiocyanate concentration and BMDs using data from the 2011–2018 National Health and Nutrition Examination Survey (NHANES). Meanwhile, we employed a widely available mixed-method, quantile g-computation (qgcomp), to evaluate the integrated effect of perchlorate, nitrate, and thiocyanate exposures with BMDs.

## 2. Methods

### 2.1. Study Population

**Figure 1:**
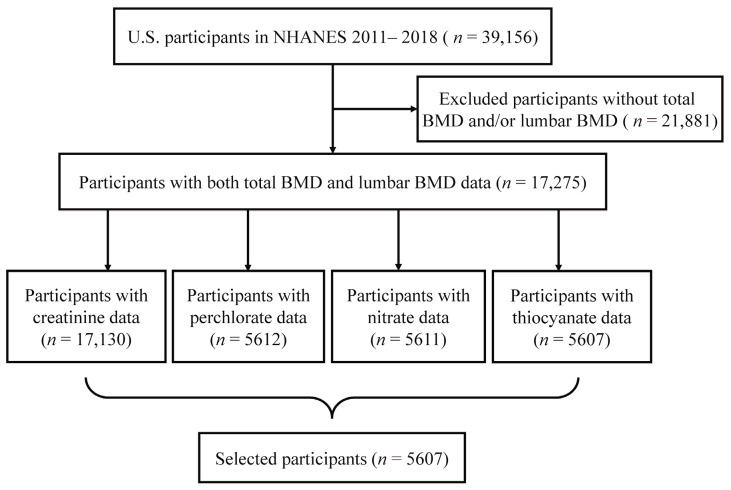
Flow diagram of participant selection in NHANES 2011–2018. Abbreviations: NHANES, National Health and Nutrition Examination Survey; BMD, bone mineral density.

The data we used came from the 2011–2018 NHANES, a complex, multi-stage, probability sampling of US people. There were 39,156 participants initially enrolled. Among them, 21,881 participants without data on the BMD examination were excluded. Additionally, 16,274 participants were excluded without measurement data on perchlorate, nitrate, thiocyanate, and creatinine. Finally, 5607 participants were enrolled in this analysis (Figure 1).

### 2.2. Exposure Measurements

Urine samples were submitted at the Mobile Examination Center and were stored at −20 °C after collection until further processing. The levels of perchlorate, nitrate, and thiocyanate in urine were evaluated using ion chromatography coupled with electrospray tandem mass spectrometry. Urine perchlorate, nitrate, and thiocyanate had lower limits of detection (LODs) of 0.05, 700, and 20 ng/mL, respectively.

All exposures were detected above 90%, and the square root of 2 was used to calculate values below the LOD. The NHANES Laboratory Procedures Handbook is located on the web: https://wwwn.cdc.gov/nchs/nhanes/default.aspx (accessed on 20 January 2024).

### 2.3. BMD Measurement

Hologic Discovery Model A densitometers measure total and lumbar BMD using dual-energy X-ray absorption. More details on the BMD examination are available in the DXXLSA dataset on the NHANES website.

### 2.4. Covariates

Based on prior research, we selected potential confounding covariates related to BMD. Thus, the adjusted model was constructed through the following variables: age, gender (male and female), race (Mexican American, non-Hispanic White, non-Hispanic Black, and other), BMI (normal weight, overweight, and obesity), family PIR (<1 and ≥1), an education level (under high school, high school or equivalent, and above high school), serum cotinine (≥10 ng/mL, 1–9.9 ng/mL, and <1 ng/mL), exercise (<10 min per week and ≥10 min per week), serum 25 (OH) D, thyroid problem (yes or no), diabetes (yes or no), and hypertension (yes or no). For categorical variables, missing data for the covariates were assigned as a missing indicator category, and the missing data for continuous variables were imputed as their median.

The body mass index (BMI) was determined by dividing the measured body mass by the square of the measured height. For adults, the BMI was categorized as follows: normal weight (<25 kg/m^2^), overweight (25 to <30 kg/m^2^), or obesity (>30 kg/m^2^) [28]. The BMI categories for children were classified as follows: normal (<28.97 kg/m^2^), overweight (28.97 to <34.57 kg/m^2^), or obese (>34.57 kg/m^2^), based on the age- and gender-specific criteria established by international research [29]. Hypertension was defined based on a diagnosis from a physician, current usage of the medicine to treat hypertension, an average systolic blood pressure of 140 or higher, or a diastolic blood pressure of 90 mm Hg or higher. Diabetes was defined based on a diagnosis from a physician, current usage of drugs that lower blood sugar levels, self-reported current usage of insulin, or an HbA1c level above 6.5%. Detailed means of acquiring and analyzing these variables can be obtained at https://wwwn.cdc.gov/nchs/nhanes/ (accessed on 20 January 2024).

### 2.5. Statistical Analysis

Natural logarithms (ln) were computed when perchlorate, nitrate, and thiocyanate levels were handled as continuous variables and converted in order to enhance statistical models for data normality, as these variables had a right-skewed distribution. In order to account for urinary dilution when assessing perchlorate, nitrate, and thiocyanate concentrations, we adjusted these concentrations based on urine creatinine levels. The major relationships between exposures, outcomes, and covariates were presented in the directed acyclic graphs (Figure S1). In this study, we presented the concentrations of perchlorate, nitrate, and thiocyanate using both the geometric mean with its 95% confidence interval (CI) and the median with its 95% CI.

The relationships between urine perchlorate, nitrate, and thiocyanate were determined using Spearman correlation coefficient calculations. The relationships between urine perchlorate, nitrate, and thiocyanate in urine and BMDs were evaluated using a survey-weighted generalized linear model (GLM). We constructed two models to evaluate the impact of perchlorate, nitrate, and thiocyanate metabolites: Model 1 included the following covariates: age, gender, BMI, race, income to poverty ratio, education, serum cotinine levels, drinking, exercise, serum 25 (OH) D, thyroid problems, hypertension, and diabetes. Model 2 included all covariates in Model 1 plus additional covariates: urinary perchlorate, nitrate, and thiocyanate levels. Using restricted cubic splines (RCSs), urine perchlorate, nitrate, and thiocyanate levels were evaluated nonlinearly in relation to BMDs.

Additionally, we categorized urinary perchlorate, nitrate, and thiocyanate in tertiles to explore potential dose-response relationships. To assess the effect of urine perchlorate, nitrate, and thiocyanate on BMD levels, both single and combined quantile-based g-computation methods (qgcomp) were used. Qgcomp is a recently introduced way that analyzes the combined impact of mixture components by creating weighted indices. Qgcomp does not need the directional homogeneity assumption, and it assesses the cumulative impact of increasing one quantile’s overall exposure. These weights show the percentage contribution of each exposure variable to the impact, which were then summed to either 1 or −1. Furthermore, age, gender, and BMI were stratified, and interaction variables were included in the regression models to estimate effect adjustments.

To ensure the reliability and validity of the results of this study, sensitivity analyses were also performed to assess the effect of other variables on the relation between the urine perchlorate, nitrate, and thiocyanate levels and BMD. First, we examined whether our results were sensitive to influential observations by removing extreme values of urine perchlorate, nitrate, thiocyanate, and creatinine. A value that falls below or is above the 99th percentile is defined as an extreme value. Second, due to a variety of factors influencing BMD with age, sex, and race, we categorized the participants into two distinct groups based on age: ≥20 years old and less than 20 years old. Lumber BMD Z-scores were used to analyze the association between the concentrations of perchlorate, nitrate, and thiocyanate concentrations and BMD in participants less than 20 years old [30]; Lumber BMD T-scores were used to analyze the association between the concentrations of perchlorate, nitrate, and thiocyanate concentrations and BMD in participants aged 20 years or older [31,32].

R (version 4.2.1) and STATA (version 17.0) were used for all statistical analyses. The R package “qgcomp” was utilized to implement the qgcomp model [28,29]. It was considered statistically significant if *p* > 0.05 was double-sided.

## 3. Results

### 3.1. Baseline Characterization

Table 1 presents the demographic characteristics of the study participants. Of the 5607 participants, 49.0% were female, 1309 (16.2%) were below the poverty level, 2042 (48.2%) were above high school, 2948 (45.6%) were normal weight, 810 (19.4%) had hypertension, 838 (22.0%) had hyperlipidemia, 368 (6.3%) had diabetes, 1713 (37.8%) were physically active, and 213 (7.5%) had been told they had a thyroid problem. Children and adolescents accounted for nearly 25% of the study population. Non-Hispanic whites made up the majority of the participants (58.6%). The median levels of serum 25 (OH) D, total BMD, and lumbar BMD were 61.70 (50.20, 78.06) nmol/L, 1.09 (1.00, 1.16) g/cm^2^, and 1.00 (0.89, 1.11) g/cm^2^, respectively. Based on the tertiles of urine perchlorate, nitrate, and thiocyanate, Table S1 displayed the characteristics of the study population. Female individuals had higher urine concentrations of perchlorate and nitrate (Table S1). Perchlorate, nitrate, and thiocyanate levels were higher in participants who weighed normal (Table S1).

### 3.2. Distribution of Perchlorate, Nitrate, and Thiocyanate

**Figure 2:**
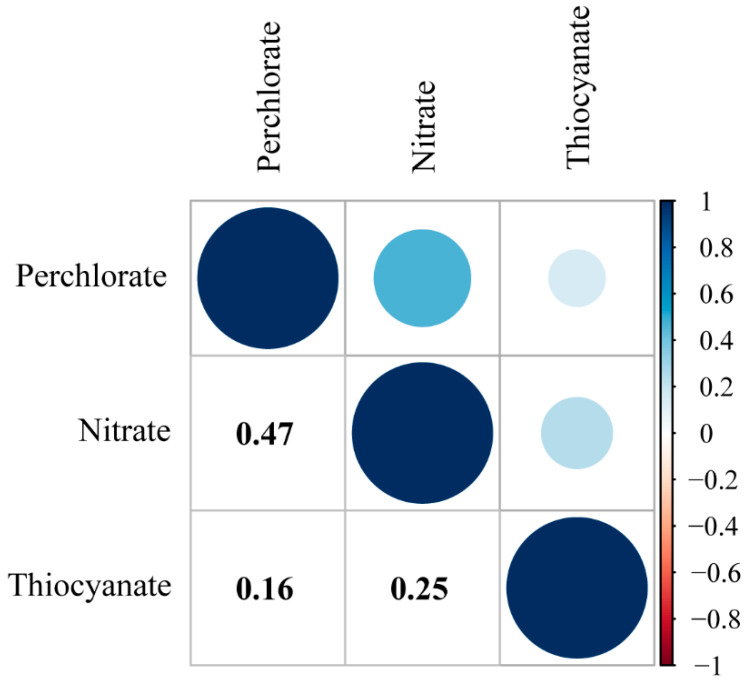
Spearman rank correlation matrix for urinary perchlorate, nitrate, and thiocyanate measured in the population (*n* = 5607), NHANES 2011–2018.

Table 2 shows the distributions of perchlorate, nitrate, and thiocyanate concentrations. The three exposures were detected at a rate higher than 99% (Table 2). The median (25th, 75th percentile) concentrations of creatinine-adjusted nitrate, thiocyanate, and perchlorate were 44.73 (32.90, 63.58) mg/g, 1.05 (0.57, 2.01) mg/g, and 2.59 (1.60, 4.36) μg/g, respectively. Correlation coefficient matrices are shown in Figure 2. Across all groups, the three biomarker associations were below 0.5 (ranging from 0.16 to 0.47) (Figure 2).

### 3.3. Urinary Perchlorate, Nitrate, and Thiocyanate in Correlation with BMDs

**Figure 3:**
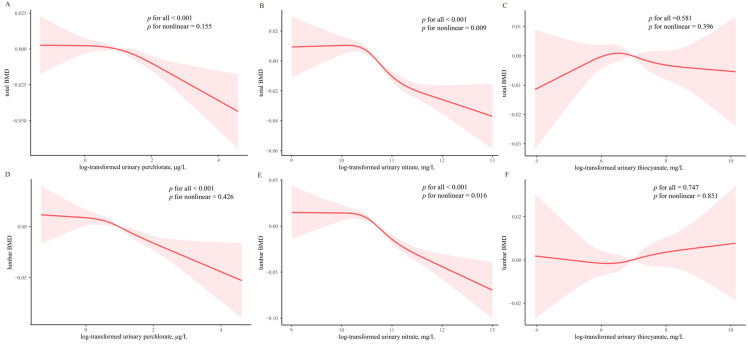
Predicted cubic splines for the associations of urinary perchlorate, nitrate, and thiocyanate with BMDs. The relationship between total BMD and urinary perchlorate (**A**), nitrate (**B**), and thiocyanate (**C**). The relationship between lumbar BMD and urinary perchlorate (**D**), nitrate (**E**), and thiocyanate (**F**). Models were adjusted for age, gender, body mass index, race, income to poverty ratio, education, serum cotinine levels, drinking, exercise, serum 25 (OH) D, thyroid problems, hypertension, diabetes, urinary perchlorate, nitrate, and thiocyanate levels. Abbreviations: BMD, bone mineral density; CI, confidence interval; *p* for trend of urinary perchlorate, nitrate, and thiocyanate.

Table 3 shows the relationship between each chemical exposure and BMD. In Model 1, each unit rise in perchlorate levels (ln-transformed) was negatively correlated with total BMD (β = −0.008; 95% CI: −0.013, −0.003; *p* < 0.001) and lumbar BMD (β = −0.013; 95% CI: −0.020, −0.007; *p* < 0.001), and each unit increase in nitrate levels was also adversely associated with total BMD (β =−0.022; 95% CI: −0.029, −0.014; *p* < 0.001) and lumbar BMD (β = −0.031; 95% CI: −0.042, −0.021; *p* < 0.001). The total BMD (β = −0.002; 95% CI: −0.005, 0.002; *p* = 0.314) and lumbar BMD (β = 0.001; 95% CI: −0.005, 0.006; *p* = 0.705) did not significantly correlate with higher urinary thiocyanate concentrations. Additionally, the models incorporated the tertiles of perchlorate, nitrate, and thiocyanate concentrations. In Model 1, compared to the lowest perchlorate concentration tertile, the total BMD showed a percent change, and the 95% CI of total BMD were −0.013 (−0.022, −0.004) for T3 and lumbar BMD were −0.025 (−0.038, −0.012) for T3. Tertiles analyses also showed that nitrate was negatively correlated with total BMD (the third tertiles vs. the first tertiles: −0.030, 95% CI, −0.020 to −0.007; *p* < 0.001) and lumbar BMD (the third tertiles vs. the first tertiles: −0.044, 95% CI, −0.058 to −0.030; *p* < 0.001). After further mutually adjusting for perchlorate, nitrate, and thiocyanate (model 2), BMDs were significantly associated with perchlorate and nitrate per unit increase, with adjusted percent change of −0.003 (95% CI: −0.007, −0.002, *p* = 0.018) and −0.021 (95% CI: −0.029, −0.013, *p* < 0.001) for total BMD, and −0.008 (95% CI: −0.015, −0.001, *p* = 0.018) and −0.032 (95% CI: −0.043, −0.021, *p* < 0.001) for lumbar BMD, respectively. Negative correlations between nitrate and total BMD were seen in the third group (β = −0.030; 95% CI: −0.040, −0.019; *p* < 0.001), as was a negative correlation with lumbar spine BMD (β = −0.043; 95% CI: −0.058, −0.027; *p* < 0.001). Additionally, the RCS results demonstrated no negative nonlinear dose-response correlation between the urine perchlorate and total BMD (nonlinear *p* = 0.155) and lumbar spine BMD (nonlinear *p* = 0.426) (Figure 3A,B). A negative nonlinear dose–response relationship exists between urinary nitrate and total BMD (nonlinear *p* = 0.009) and lumbar BMD (nonlinear *p* = 0.016) (Figure 3C,D).

### 3.4. Relation of Urinary Perchlorate, Nitrate, and Thiocyanate with BMD by Qgcomp Analysis

**Figure 4:**
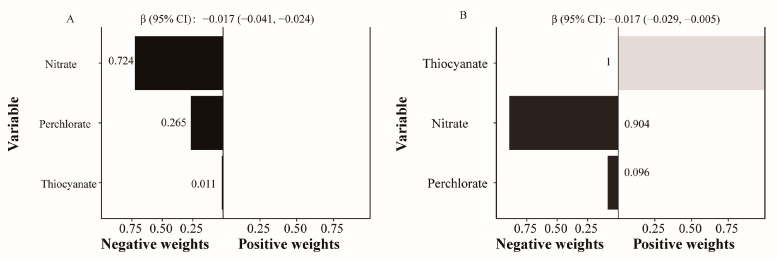
Association of co-exposure of perchlorate, nitrate, and thiocyanate with total BMD (**A**) and lumbar BMD (**B**) by qgcomp models. Models were adjusted for age, gender, body mass index, race, income to poverty ratio, education, serum cotinine levels, drinking, exercise, serum 25 (OH) D, thyroid problems, hypertension, diabetes, urinary perchlorate, nitrate, and thiocyanate levels. Abbreviations: BMD, bone mineral density; CI, confidence interval.

Figure 4 shows the results of the combined effects of perchlorate, nitrate, and thiocyanate assessment by qgcomp analysis. Urinary perchlorate, nitrate, and thiocyanate in the mixture were negatively linked to total BMD (β −0.017; 95% CI (−0.041, −0.024); *p* < 0.05) and lumbar spine BMD (β −0.017; 95% CI (−0.029, −0.005); *p* < 0.05). In line with our previous result, perchlorate and nitrate were negatively correlated with total BMD and lumbar BMD (sum of negative coefficients 0.989 and 1, respectively). Nitrate made the most negative contribution to total BMD (72.4%) and to lumbar BMD (90.4%).

### 3.5. Subgroup Analysis

**Figure 5:**
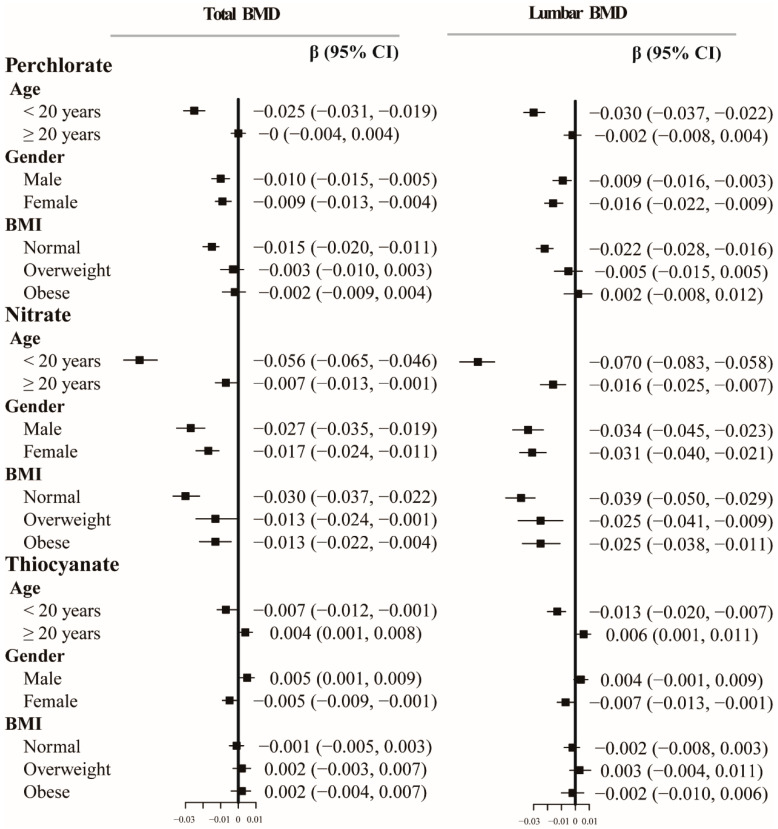
Regression coefficients and 95% confidence intervals (95% CIs) between BMDs with urinary perchlorate, nitrate, and thiocyanate concentrations in NHANES 2011–2018, stratified by subgroups. Models were adjusted for age, gender, body mass index, race, income-to-poverty ratio, education, serum cotinine levels, drinking, exercise, serum 25 (OH) D, thyroid problems, hypertension, and diabetes, except for the stratified variable. Abbreviations: BMD, bone mineral density; CI, confidence interval.

Figure 5 displays the results of the stratified analysis. Higher concentrations of perchlorate and thiocyanate were generally associated with lower BMDs in most subgroups, which aligns with the primary result. Perchlorate, nitrate, and thiocyanate exposures and BMDs were correlated when stratified by age (Figure 5). Notably, only children and adolescents (under 20 years old) demonstrated a significantly negative correlation between BMDs and urinary perchlorate and thiocyanate (each *p*-value is less than 0.001). Specifically, there were no significant differences of total BMD and lumbar BMD between the children and adolescents group (β = −0.025, 95% CI: −0.031, −0.019; β = −0.030, 95% CI: −0.037, −0.022), male group (β = −0.010, 95% CI: −0.015, −0.005; β = −0.009, 95% CI: −0.016, −0.003), female group (β = −0.009, 95% CI: −0.013, −0.004; β = −0.016, 95% CI: −0.022, −0.009), and normal weight group (β = −0.015, 95 %CI: −0.020, −0.011; β = −0.022, 95% CI: −0.028, −0.016). In the children and adolescents group and the female group, each unit increase in thiocyanate was associated with total BMD and lumbar BMD, with adjusted β coefficients of −0.007 (95% CI: −0.012, −0.001) and −0.005 (95% CI: −0.009, −0.001) for total BMD, and −0.013 (95% CI: −0.020, −0.007) and −0.007 (95% CI: −0.013, −0.001) for lumbar BMD, respectively. The relationship between nitrate and BMD did not vary considerably among groups of different ages, sexes, and BMI.

### 3.6. Sensitivity Analyses

Generally, the results from the sensitivity analysis were equivalent to the main results. When excluding participants with extreme values, negative associations with BMD were also observed for perchlorate and nitrate exposure, with an adjusted β coefficient of −0.001 (95% CI: −0.006, 0.004, *p*-value = 0.735) and −0.023 (95% CI: −0.031, −0.015, *p*-value < 0.001) for total BMD, and −0.008 (95% CI: −0.015, −0.001, *p*-value = 0.046) and −0.035 (95% CI: −0.047, −0.022, *p*-value < 0.001) for lumbar BMD, respectively (Table S2). The above results show that the use of different BMD definitions did not have a significant impact on the relationship between perchlorate, nitrate, and thiocyanate exposures and BMD, reflecting the robustness of our results.

## 4. Discussion

To our knowledge, this is the first and largest general population-based study exploring BMD associations with co-exposure to perchlorate, nitrate, and thiocyanate. Our results showed that perchlorate and nitrate levels were negatively correlated with BMD. qgcomp analysis showed that combined exposure to a mixture of perchlorate, nitrate, and thiocyanate was negatively correlated with total BMD, with nitrate being the main contributor to the mixture effect. We found that children and adolescents are the most sensitive subgroups whose BMD values are easily disturbed. A significant negative correlation was found between urinary perchlorate, nitrate, and thiocyanate and BMD in children and adolescents. Interestingly, the overall effects of perchlorate on normal weight were more pronounced than for overweight and obese.

Exposure to perchlorate, nitrate, and thiocyanate is widespread in the environment and diet, and the health effects of these three chemicals have been widely reported. Previous research has mostly examined the underlying relationships between perchlorate, nitrate, and thiocyanate exposed and the diverse metabolic disorders, including metabolic syndrome (MetS) [17], obesity [33], nonalcoholic fatty liver disease [34], and cardiovascular diseases [35]. There has been much attention in recent years to the effects of exposure to thiocyanate, nitrate, and perchlorate on thyroid function, but little attention has been paid to the effects of perchlorate, nitrate, and thiocyanate, either alone or in combination, on BMD. Yan et al. reported similar results to ours, namely a negative correlation between BMD and high nitrate concentrations, and nitrate accounted for the majority of reduced BMD after multivariable adjustment [26]. This is broadly consistent with our qgcomp observations. According to Xu et al., thiocyanate and nitrate were inversely associated with oral BMD [36]. Here, our results of survey-weighted generalized linear regression reported a null association between urinary thiocyanate and BMDs. In spite of the fact that our study found an association between perchlorate, nitrate, and thiocyanate with BMD, the mechanisms underlying them need to be further confirmed. Several studies might offer a plausible explanation for their mechanism.

Perchlorate is a typical endocrine disruptor for the thyroid. The RCS results showed no nonlinear relationship between perchlorate and BMDs. Studies have shown that perchlorates and nitrates are closely related to multiple thyroid indicators, which include thyroid-stimulating hormone (TSH), free thyroxine (fT4), and free triiodothyronine (fT3). Triiodothyronine (T3) acts on the thyroid hormone receptor alpha (TR-α) of osteoblasts and osteoclasts [37]. Osteoblasts can increase the formation of osteoblasts and bone, while osteoclasts can increase the process of osteoclasts and bone resorption [37]. TSH can inhibit local tumor necrosis factor-alpha (TNF-α) production and reduce the incidence of bone formation, which is one of the key regulators of bone metabolism. Previous studies have shown that regardless of whether TSH ranges from below normal to above normal range, it would diminish BMD levels [38,39]. Animal experiments have revealed that hypothyroidism results in the almost complete arrest of bone remodeling, and bone turnover in hypothyroid mice is reduced [40]. In addition, thyroid hormones can also cooperate with multiple endocrine hormones, such as calcitonin, prolactin, growth hormone, and sex hormones (estrogen and androgen), to participate in the whole body’s bone metabolism process, including bone growth, development and maturation, bone reconstruction, bone absorption, calcium, and phosphorus metabolism [40]. Additionally, the study by Han et al. showed that perchlorate, nitrate, and thiocyanate are strongly correlated to testosterone levels and observed the opposite correlations in agreement between perchlorate and thiocyanate [18]. Perchlorate may alter the oxidative stress state of testicular tissues, thereby impeding testosterone secretion and production [18]. Amory et al. have found that plasma-free testosterone levels were positively associated with an increase in BMD [41]. In vitro experiments, testosterone can inhibit the proliferation and activity of osteoclasts, stimulate the apoptosis of osteoclasts, and lead to the shortening of the lifespan of osteoclasts, thereby reducing bone resorption [42].

In the present study, we reported that nitrate was negatively associated with BMDs. Multiple sensitivity analyses showed that these results remained robust. Qgcomp indicated a negative association between nitrate and BMD, and a nonlinear relationship between nitrate exposure and BMD was noted in the RCS. However, previous findings exploring the association between nitrate and BMDs had conflicting results. A prospective study of 6201 elderly women showed that the intermittent administration of nitrates may enhance BMD [43]. A randomized controlled trial reported that there was no relationship between nitrates and BMD, while Yan et al. [26] and Shiue et al. [36] studies have shown that nitrates can cause a decrease in bone density. Dietary intake is the main source of nitrates in the body, and excessive intake, especially of vegetables and water contaminated with nitrates, may be associated with reduced BMD [44] because nitrates are easily transformed into nitric oxide (NO) in the body [45]. NO is a highly bioactive molecule that has a biphasic, concentration-dependent effect on the skeletal system and plays an important role in the overall formation process of BMDs [46]. High concentrations of NO (caused by iNOS activation or exogenous high dosages of NO donors) may cause bone loss, whereas low concentrations of NO (owing to eNOS activity) are linked to osteocyte and osteoblast activity, mediating osteoclast bone absorption [47]. A retrospective cohort study reported that NO exposure was linked to hospital admissions due to hip fractures [48]. Likewise, a population-based retrospective cohort study reported that exposure to NO was linked to a higher incidence of osteoporosis [49]. In addition, studies in animals have shown that nitrates may change the concentration of circulating neutral steroids, particularly androgens [50]. The hormone is thought to impact osteoclastic activity indirectly by reducing the production and release of receptor activators of nuclear factor κB ligand (RANKL) in neighboring osteoblasts or osteocytes [42,50]. Overall, factors such as high-nitrate vegetables, contaminated drinking water, and the conversion of nitrates in the body may affect BMD.

Our study showed that the link between co-exposure to perchlorate, nitrate, and thiocyanate and BMD varies among different populations, including adults, children, and adolescents. This might be due to the fact that children and adolescents are undergoing rapid growth and development, rendering them more vulnerable to the impact of environmental pollutants. In addition, our stratified analysis based on BMI categories revealed that perchlorate exposure was more negatively associated with BMDs in normal-weight participants than in overweight and obese participants (−0.015 vs. −0.002 and −0.003, respectively). Normal-weight participants were also more negatively correlated with nitrate and BMDs than overweight and obese participants (−0.030 vs. −0.013 and −0.013, respectively). Normal-weight participants were significantly more negatively associated with thiocyanate than overweight and obese participants (−0.001 vs. 0.002 and 0.002, respectively). Recently, a NHANES study found a significant association between BMI and BMD [51]. Song et al. indicated that a higher BMI was related to increased bone density, and the effects were similar throughout the bones [52]. Similarly, total hip and femoral neck BMD were significantly higher in obese and overweight boys compared to boys with normal BMI [53]. The relationship between BMI and BMD was significantly positive, which might be due to the mechanical protective effect of a higher BMI on bones [54]. The greater the BMI, the larger the mechanical load on the bones, resulting in stimulation of osteoblast activity and increasing bone density [54]. Therefore, urinary perchlorate, nitrate, and thiocyanate correlate differently with BMD at different BMIs, perhaps because BMI affects BMD differently.

Urinary thiocyanate was found to be positively related to BMDs in males. Diet and smoking are the main sources of exposure to thiocyanates in humans. Therefore, thiocyanate can also serve as a biomarker of cyanide intake via smoking or diet [55,56]. Thiocyanate exposure has been linked to other health issues outside affecting thyroid hormone production, including neurodevelopment [57], stroke [58], chronic kidney disease (CKD) [59], oral health [60], and cardiovascular disease [35]. Nevertheless, thiocyanate also offers certain health benefits, like lowering blood pressure, preventing myocardial ischemia-reperfusion injury [61], improving vascular endothelial function [62], and enhancing the respiratory tract [63]. There is limited research on the impact of thiocyanate on BMDs. Nevertheless, a study of middle-aged and elderly men found a positive correlation between total testosterone and BMD [64]. A cross-sectional study of 6201 children and adolescents in the US disclosed that exposure to thiocyanate was positively correlated with total testosterone (TT) levels [18]. Therefore, this suggested that urinary thiocyanate may be correlated with BMD in male adults due to TT concentration, which merits further investigation. Furthermore, it is noteworthy that the complexity of thiocyanate sources presents a dual impact on bone health. On the one hand, thiocyanates can be ingested from green vegetables [65], potentially conferring benefits to bone health [66]. On the other hand, thiocyanates can be ingested from tobacco [67], which may adversely affect bone homeostasis [68]. This factor may contribute to the differences in the association between thiocyanate and BMD by gender. To sum up, the difference in the relationship between thiocyanate levels and BMD between different genders is the result of a combination of multiple factors, which need to be considered in conjunction with hormone levels, lifestyle habits, and other factors. Further research may help to better understand these complex mechanisms and relationships.

Several strengths of this research are worth mentioning. A first attempt has been made to investigate the connection between BMDs with co-exposure to perchlorate, nitrate, and thiocyanate. This study explored the possible connection between these three substances and BMDs for further investigation into the underlying physiological processes. Additionally, we performed sensitivity analysis using Z-scores BMD for adolescents and T-scores BMD for adults, which makes the results robust. Lastly, we examined the combined effects of perchlorate, nitrate, and thiocyanate mixtures on BMD using qgcomp models. It is not uncommon for people in real life to be exposed to multiple pollutants at once, so this statistical approach can help us understand mixing effects and draw more accurate conclusions. In spite of this, there are also some obvious limitations. First, despite controlling for many potential covariates, residual confounding still exists. For example, we did not have access to data on the participants’ diet exposures and occupations. Therefore, we could not distinguish the specific sources of perchlorate, nitrate, and thiocyanate in our bodies. Second, urine samples may not adequately represent long-term exposures since they are collected at a single point in time. Time measurements of 24 h or more are ideal, but they would be difficult to enforce because of the large population. Third, because of the observational character of cross-sectional studies, causality cannot be inferred.

## 5. Conclusions

In summary, our research showed a negative correlation between BMDs and perchlorate and nitrate in the US general population. The BMDs are inversely correlated with co-exposure to perchlorate and nitrate, with children and adolescents and the normal-weight population more susceptible. Further studies are warranted to corroborate our findings and explore underlying mechanisms that are important to protecting the human body.

## Figures and Tables

**Table 1:** Basic demographics of the study sample participating in NHANES 2011–2018 (*n* = 5607).

Characteristic	*n* (Unweighted) (%)/Median (IQR)
Age (%)	
<20 years	2179 (25.4)
≥20 years	3428 (74.6)
Gender (%)	
male	2887 (51.4)
Female	2720 (48.6)
Race (%)	
Non-Hispanic White	1740 (58.6)
Mexican American	1013 (12.2)
Non-Hispanic Black	1219 (12)
Others	1635 (17.3)
PIR (%)	
<1	1309 (16.2)
≥1	4298 (83.8)
Education (%)	
Above high school	2042 (48.2)
High school or equivalent	879 (18.2)
Under high school	2686 (33.5)
Serum cotinine (%)	
<1.0	3882 (73.0)
1.0–9.9	306 (5.0)
≥10	1004 (22.0)
BMI (%)	
Normal	2948 (45.6)
Overweight	1276 (26.4)
Obese	1364 (28)
Hypertension: yes, *n* (%)	801 (19.4)
Hyperlipidemia: yes, *n* (%)	838 (22.0)
Diabetes: yes, *n* (%)	368 (6.3)
Exercise: yes, *n* (%)	1713 (37.8)
Thyroid problem: yes, *n* (%)	213 (7.5)
Serum 25 (OH) D (nmol/L)	61.70 (50.20, 78.06)
Total BMD (g/cm^2^)	1.09 (1.00, 1.16)
Lumbar BMD (g/cm^2^)	1.00 (0.89, 1.11)

Abbreviations: NHANES, National Health and Nutrition Examination Survey; BMI, body mass index; PIR, income to poverty ratio; and Serum 25 (OH) D, serum 25-hydroxyvitamin D.

**Table 2:** Urinary perchlorate, nitrate, and thiocyanate distribution, NHANES 2011–2018 (*n* = 5607).

	Detection	GM (95% CI)	Selected Percentile		
			25th	Median	75th
Unadjusted					
Perchlorate (μg/L)	99.84%	2.62 (2.56, 2.69)	1.45	2.69	4.74
Nitrate (mg/L)	99.79%	45.52 (44.62, 46.44)	28.10	48.90	76.10
Thiocyanate (mg/L)	99.82%	1.08 (1.05, 1.11)	0.53	1.06	2.05
Creatinine-adjusted					
Perchlorate (μg/g creatinine)	99.84%	2.70 (2.64, 2.76)	1.60	2.59	4.36
Nitrate (mg/g creatinine)	99.79%	46.84 (46.17, 47.53)	32.90	44.73	63.58
Thiocyanate (mg/g creatinine)	99.82%	1.11 (1.08, 1.14)	0.57	1.05	2.01

Abbreviations: GM: geometric mean; CI, confidence interval.

**Table 3:** Association between urinary perchlorate, nitrate, and thiocyanate levels with BMDs.

	Total BMD				Lumbar BMD			
	β (95% CI) ^a^	*p*-Value	β (95% CI) ^b^	*p*-Value	β (95% CI) ^a^	*p*-Value	β (95% CI) ^b^	*p*-Value
Perchlorate								
Per 100% increase	−0.008 (−0.013, −0.003)	<0.001	−0.003 (−0.007, −0.002)	0.018	−0.013 (−0.020, −0.007)	<0.001	−0.008 (−0.015, −0.001)	0.018
Tertiles								
T1	Reference		Reference		Reference		Reference	
T2	−0.005 (−0.013, 0.004)	0.299	0.001 (−0.008, 0.010)	0.834	−0.016 (−0.029, −0.003)	<0.001	−0.009 (−0.021, 0.004)	0.195
T3	−0.013 (−0.022, −0.004)	0.004	−0.003 (−0.012, 0.007)	0.560	−0.025 (−0.038, −0.012)	<0.001	−0.011 (−0.025, 0.003)	0.111
Nitrate								
Per 100% increase	−0.022 (−0.029, −0.014)	<0.001	−0.021 (−0.029, −0.013)	<0.001	−0.031 (−0.042, −0.021)	<0.001	−0.032 (−0.043, −0.021)	<0.001
Tertiles								
T1	Reference		Reference		Reference		Reference	
T2	−0.017 (−0.025, −0.008)	<0.001	−0.017 (−0.025, −0.008)	<0.001	−0.028 (−0.040, −0.016)	<0.001	−0.027 (−0.040, −0.015)	<0.001
T3	−0.030 (−0.020, −0.007)	<0.001	−0.030 (−0.040, −0.019)	<0.001	−0.044 (−0.058, −0.030)	<0.001	−0.043 (−0.058, −0.027)	<0.001
Thiocyanate								
Per 100% increase	−0.002 (−0.005, 0.002)	0.314	0.002 (−0.002, 0.005)	0.334	0.001 (−0.005, 0.006)	0.705	0.005 (−0.001, 0.011)	0.066
Tertiles								
T1	Reference		Reference		Reference		Reference	
T2	0.005 (−0.004, 0.014)	0.250	0.009 (0.001, 0.017)	0.046	0.004 (−0.009, 0.016)	0.570	0.010 (−0.002, 0.022)	0.115
T3	−0.005 (−0.014, 0.004)	0.286	0.002 (−0.006, 0.011)	0.586	−0.001 (−0.014, 0.011)	0.850	0.011 (−0.002, 0.023)	0.094

Model 1 (^a^) was adjusted for age, gender, body mass index, race, income to poverty ratio, education, serum cotinine levels, drinking, exercise, serum 25 (OH) D, thyroid problems, hypertension, and diabetes; Model 2 (^b^) was adjusted for model 1 + urinary perchlorate, nitrate, and thiocyanate levels. Abbreviations: BMD, bone mineral density; CI, confidence interval; *p* for trend across tertiles of urinary perchlorate, nitrate, and thiocyanate.

## Data Availability

The datasets used and/or analyzed during the current study are available from https://www.cdc.gov/nchs/nhanes/nhanes_questionnaires.htm (accessed on 20 January 2024).

## Supplementary Materials

The following supporting information can be downloaded at: https://www.mdpi.com/2072-6643/16/16/2658/s1. Figure S1: Directed acyclic graphs showing hypothetical causal relationships regarding chemical exposure and BMD in this study. Boxes with solid outlines represent variables observed in this study, and those with dashed outlines represent variables not observed. Table S1: Characteristics of study participants according to tertiles of urinary perchlorate, nitrate, and thiocyanate levels in the NHANES from 2011 to 2018 (*n* = 5607); Table S2: Associations of perchlorate, nitrate, and thiocyanate concentration with BMDs, after excluding extreme values (defined as <1st percentile or >99th percentile) of urinary perchlorate, nitrate, thiocyanate, and creatinine; Table S3: Sensitivity analyses of the regression coefficients and 95% confidence intervals (95% CI) in lumber BMD Z-scores (<20 year) and T-scores (≥20 year) in urinary perchlorate, nitrate, and thiocyanate concentrations in NHANES 2011–2018.

## Abbreviations

ANOVA, one-way analysis of variance; BMI, body mass index; BMD, bone mineral density; CI, confidence interval; CKD, chronic kidney disease; fT4, free thyroxine; fT3, free triiodothyronine; GLM, survey-weighted generalized linear model; LOD, lower limits of detection; NO, nitric oxide; NHANES, National Health and Nutrition Examination Survey; NIS, inhibitors of the sodium/iodide symporter; NCHS, National Center for Health Statistics; OP, osteoporosis; PIR, income-to-poverty ratio; Qgcomp, quantile-based g-computation; RANKL, release of receptor activators of the nuclear factor κB ligand; RCSs, restricted cubic splines; Serum 25 (OH) D, serum 25-hydroxyvitamin D; SEs, standard errors; TT, total testosterone; TSH, thyroid-stimulating hormone; T3, triiodothyronine; TR-α, thyroid hormone receptor alpha; TNF-α, tumor necrosis factor α.
