# Supplementary material for: Associations of Perchlorate, Nitrate, and Thiocyanate with Bone Mineral Density in the US General Population: A Multi-Cycle Study of NHANES 2011–2018
Source: Nutrients. 2024 Aug 11;16(16):2658. doi: 10.3390/nu16162658 (PMC11487404; doi:10.3390/nu16162658)
Supplement: Supplementary file 1 [file nutrients-16-02658-s001.zip › nutrients-3086710-supplementary.pdf]

Associations of Perchlorate, Nitrate, and Thiocyanate with Bone Mineral Density in the US General Population: A Multi-Cycle Study of NHANES 2011–2018

Donglan Wang [1,†], Ying Zhang [1,†], Yayu He [1], Fengmei Song [1], Yan Tang [1], Limou Chen [1], Yangcan Wang [1], Fei Yang [1,2,*] and Xueqiong Yao [1,3,*]

Affiliations

[1] School of Public Health, Hengyang Medical School, University of South China, Hengyang 421009, China; yayu_he@163.com (Y.H.)

[2] Hunan Provincial Key Laboratory of Clinical Epidemiology, Department of Social Medicine and Health Management, Xiangya School of Public Health, Central South University, Changsha 410000, China

[3] Laboratory of Ecological Environment and Critical Human Diseases Prevention of Hunan Province, School of Basic Medical Sciences, Hengyang Medical School, University of South China, Hengyang 421009, China

* Correspondence: phfyang@csu.edu.cn (F.Y.); yxq1212@usc.edu.cn (X.Y.)

† These authors contributed equally to this work.

† These authors contributed equally to this work.

Supporting Information

Table S1 Characteristics of study participants according to tertiles of urinary perchlorate, nitrate, and thiocyanate levels in the NHANES from 2011 to 2018 (*n* = 5607)

Table S2 Associations of perchlorate, nitrate, and thiocyanate concentration with BMDs, after excluding extreme values (defined as < 1st percentile or > 99th percentile) of urinary perchlorate, nitrate, thiocyanate, and creatinine.

Table S3 Sensitivity analyses of the regression coefficients and 95% confidence intervals (95% CI) in lumber BMD Z-scores (< 20 year) and T-scores (≥ 20 year) in urinary perchlorate, nitrate, and thiocyanate concentrations in NHANES 2011–2018.

Figure S1 Directed acyclic graphs showing hypothetical causal relationships regarding chemical exposure and BMD in this study. Boxes with solid outlines represent variables observed in this study, and those with dashed outlines represent variables not observed.

Table S1 Characteristics of study participants according to tertiles of urinary perchlorate, nitrate, and thiocyanate levels in the NHANES from 2011 to 2018 (n = 5607)

Characteristic	Perchlorate (μg/L)				Nitrate (mg/L)				Thiocyanate (mg/L)			
	T1	T2	T3	*p*	T1	T2	T3	*p*	T1	T2	T3	*p*
Age				< 0.001				0.01				< 0.001
<20 years	21.2	25.1	29.6		20.2	28.7	27.3		28.0	29.6	19.7	
≥20 years	78.8	74.9	70.4		79.8	71.3	72.7		72.0	70.4	80.3	
Gender				0.01				< 0.001				0.60
Female	42.2	50.9	52.3		37.9	50.3	57.7		47.0	49.2	49.4	
male	57.8	49.1	47.7		62.1	49.7	42.3		53.0	50.8	50.6	
Race				< 0.001				< 0.001				< 0.001
Mexican American	11.1	12.4	13		12.8	12.4	11.3		15.3	13.7	8.3	
Non-Hispanic Black	20.1	10.1	6.4		20.2	10.8	5.0		13.9	11.8	10.6	
Non-Hispanic White	49.8	61.9	63.3		50.8	59.8	65.1		48.8	57.0	67.7	
Others	19.1	15.6	17.4		16.2	17	18.7		22.0	17.4	13.4	
PIR				0.02				0.20				0.06
< 1	18.2	16.6	14		17.4	16.5	14.9		16.7	14.2	17.7	
≥ 1	81.8	83.4	86		82.6	83.5	85.1		83.3	85.8	82.3	
Education				0.07				0.01				0.04
Above high school	48.5	47.2	49.1		49	45.1	50.6		46.7	51.2	47	
High school or equivalent	21.1	19.7	13.9		23.1	17.7	13.9		17.7	15.4	21.1	
Under high school	30.4	33.1	37		28.0	37.3	35.4		35.6	33.4	31.9	
Serum cotinine				0.01				0.20				< 0.001
< 1.0	65.6	75.1	77.6		73.3	69.8	75.9		83.9	87.2	52.2	
1.0–9.9	5.5	4.9	4.7		5.4	6.0	3.8		5.4	4.7	5.0	
≥ 10	28.9	20	17.7		21.3	24.3	20.4		10.7	8.0	42.8	
BMI				0.01				< 0.001				0.60
Normal	40.1	44.0	52.5		36.6	47.5	52.8		46.4	47.1	43.8	
Overweight	27.6	26.3	25.3		29.1	26.5	23.5		26.7	25.6	26.8	
Obese	32.3	29.6	22.2		34.2	26.1	23.7		26.9	27.3	29.4	
Hypertension: yes, *n* (%)	19.8	21	17.2	0.30	20.6	18.2	19.4	0.40	17.6	18.9	21.3	0.40
Hyperlipidemia: yes, *n* (%)	20.5	22.6	22.9	0.60	21.0	22.1	23.0	0.60	21.5	20.2	23.8	0.05
Diabetes: yes, *n* (%)	6.9	6.3	5.9	0.70	7.7	5.6	5.8	0.30	6.1	5.9	6.9	0.60

Table S1 Continue.

Exercise: yes, *n* (%)	35.5	38.2	39.6	0.40	37.3	38.5	37.6	0.80	41.1	42.8	30.8	<0.001
Thyroid problem: yes, *n* (%)	6.2	8.2	8.0	0.40	5.9	8.2	8.5	0.20	8.8	6.2	7.5	0.20
Serum 25 (OH) D (nmol/L)	59.49 (44.90, 72.01)	63.00 (51.20, 78.90)	65.81 (53.90, 81.52)	0.02	59.49 (46.30, 74.89)	61.50 (50.50, 76.90)	65.40 (53.30, 82.30)	0.03	59.70 (49.20, 75.69)	61.89 (49.85, 76.90)	63.70 (51.80, 80.62)	0.01

Data were expressed as the *n* (%) or median (IQR).

Abbreviations: NHANES, National Health and Nutrition Examination Survey; SD, standard deviation; BMI, body mass index; PIR, income to poverty ratio; Serum 25 (OH) D, serum 25-hydroxyvitamin D.

Table S2 Associations of perchlorate, nitrate, and thiocyanate concentration with BMDs, after excluding extreme values (defined as < 1st percentile or > 99th percentile) of urinary perchlorate, nitrate, thiocyanate, and creatinine.

	Total BMD				Lumbar BMD			
	β (95%CI) [a]	*p*	β (95%CI) [b]	*p*	β (95%CI) [a]	*p*	β (95%CI) [b]	*p*
**Perchlorate**								
Per 100% increase	–0.006 (–0.011, –0.001)	0.014	–0.001 (–0.006, 0.004)	0.735	–0.015 (–0.022, –0.008)	< 0.001	–0.008 (–0.015, –0.001)	0.046
Tertiles								
T1	Reference		Reference		Reference		Reference	
T2	–0.005 (–0.014, 0.004)	0.284	0 (–0.008, 0.009)	0.932	–0.018 (–0.031, –0.005)	0.007	–0.011 (–0.024, 0.002)	0.107
T3	–0.011 (–0.020, –0.002)	0.017	–0.002 (–0.011, 0.008)	0.693	–0.023 (–0.037, –0.010)	< 0.001	–0.012 (–0.026, 0.003)	0.108
**Nitrate**								
Per 100% increase	–0.023 (–0.031, –0.015)	< 0.001	–0.023 (–0.032, –0.015)	< 0.001	–0.036 (–0.048, –0.025)	< 0.001	–0.035 (–0.047, –0.022)	< 0.001
Tertiles								
T1	Reference		Reference		Reference		Reference	
T2	–0.018 (–0.026, –0.009)	< 0.001	–0.018 (–0.026, –0.009)	< 0.001	–0.029 (–0.042, –0.017)	< 0.001	–0.028 (–0.041, –0.016)	< 0.001
T3	–0.028 (–0.038, –0.018)	< 0.001	–0.028 (–0.038, –0.017)	< 0.001	–0.041 (–0.056, –0.027)	<0.001	–0.040(–0.055, –0.024)	<0.001
**Thiocyanate**								
Per 100% increase	–0.001 (–0.005,0.003)	0.480	0.002 (–0.002,0.006)	0.361	0.001 (–0.005, 0.006)	0.876	0.006 (0.001, 0.012)	0.044
Tertiles								
T1	Reference		Reference		Reference		Reference	
T2	0.005 (–0.004, 0.014)	0.250	0.008 (–0.001, 0.017)	0.067	0.004 (–0.009, 0.016)	0.575	0.009 (–0.003, 0.022)	0.140
T3	–0.004 (–0.013, 0.005)	0.397	0.003 (–0.006, 0.012)	0.581	0.001 (–0.012, 0.014)	0.908	0.011 (–0.001, 0.024)	0.080

The Model 1 ([a]) was adjusted for age, gender, body mass index, race, income to poverty ratio, education, serum cotinine levels, drinking, exercise, serum 25–hydroxyvitamin D, thyroid problems, hypertension, and diabetes;
The Model 2 ([b]) was adjusted for model 1 + urinary perchlorate, nitrate, and thiocyanate levels.

Abbreviations: BMD, bone mineral density; CI, confidence interval; *p* for trend of urinary perchlorate, nitrate, and thiocyanate.

Table S3 Sensitivity analyses of the regression coefficients and 95% confidence intervals (95% CI) in lumber BMD Z-scores (< 20 year) and T-scores (≥ 20 year) in urinary perchlorate, nitrate, and thiocyanate concentrations in NHANES 2011–2018

	< 20 year Z-scores				≥ 20 year T-scores			
	β (95%CI) [a]	*p*	β (95%CI) [b]	*p*	β (95%CI) [a]	*p*	β (95%CI) [b]	*p*
**Perchlorate**								
Per 100% increase	–0.048 (–0.120, 0.024)	0.194	–0.008 (–0.084, 0.069)	0.847	–0.054 (–0.128, 0.021)	0.157	–0.011 (–0.090, 0.068)	0.787
Tertiles								
T1	Reference		Reference		Reference		Reference	
T2	0.107 (–0.030, 0.244)	0.125	0.156 (0.017, 0.295)	0.028	–0.161 (–0.305, –0.016)	0.030	–0.125 (–0.270, 0.020)	0.092
T3	0.059 (–0.089, 0.207)	0.436	0.125 (–0.040, 0.290)	0.136	–0.107 (–0.252, 0.039)	0.151	–0.034 (–0.193, 0.125)	0.674
**Nitrate**								
Per 100% increase	–0.262(–0.382, –0.142)	<0.001	–0.270 (–0.397, –0.142)	<0.001	–0.180(–0.292, –0.067)	0.002	–0.207 (–0.329, –0.084)	0.001
Tertiles								
T1	Reference		Reference		Reference		Reference	
T2	–0.157 (–0.311, –0.003)	0.045	–0.190 (–0.351, –0.030)	0.020	–0.139 (–0.272, –0.006)	0.040	–0.142 (–0.281, –0.003)	0.046
T3	–0.167 (–0.328, –0.005)	0.043	–0.213 (–0.389, –0.036)	0.018	–0.240 (–0.400, –0.080)	0.003	–0.261 (–0.436, –0.086)	0.004
**Thiocyanate**								
Per 100% increase	–0.006 (–0.073, 0.061)	0.864	0.025 (–0.044, 0.094)	0.470	0.038(–0.021, 0.097)	0.209	0.071 (0.011, 0.132)	0.021
Tertiles								
T1	Reference		Reference		Reference		Reference	
T2	–0.032 (–0.167, 0.102)	0.635	–0.023 (–0.158, 0.112)	0.741	0.095 (–0.048, 0.238)	0.193	0.135 (–0.007, 0.277)	0.063
T3	–0.000 (–0.153, 0.153)	0.999	0.017 (–0.138, 0.173)	0.827	0.077 (–0.066, 0.221)	0.289	0.151 (0.006, 0.297)	0.042

The Model 1 ([a]) was adjusted for age, gender, body mass index, race, income to poverty ratio, education, serum cotinine levels, drinking, exercise, serum 25-hydroxyvitamin D, thyroid problems, hypertension, and diabetes;
The Model 2 ([b]) was adjusted for model 1 + urinary perchlorate, nitrate, and thiocyanate levels.

Abbreviations: NHANES, National Health and Nutrition Examination Survey; BMD, bone mineral density; CI, confidence interval; *P* for trend of urinary perchlorate, nitrate, and thiocyanate.

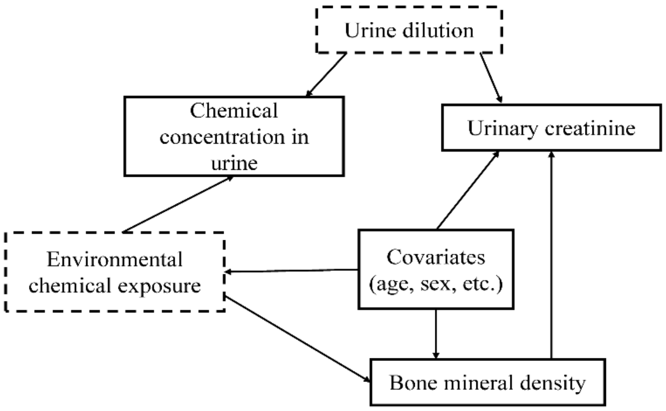

Figure S1 Directed acyclic graphs showing hypothetical causal relationships regarding chemical exposure and BMD in this study. Boxes with solid outlines represent variables observed in this study, and those with dashed outlines represent variables not observed.
